# The role of socioeconomic status and pre-pregnancy BMI in postpartum psychiatric episodes – a mediation study

**DOI:** 10.1007/s00127-026-03089-1

**Published:** 2026-04-20

**Authors:** Mette-Marie Zacher Kjeldsen, Line Bager, Katrine Holde, Trine Munk-Olsen, Liselotte Vogdrup Petersen

**Affiliations:** 1 NCRR – National Centre for Register-Based Research, Department of Public Health, Aarhus University, Aarhus, Denmark; 2 CIRRAU – Centre for Integrated Register-based Research, Department of Public Health, Aarhus University, Aarhus, Denmark; 3 Department of Clinical Research, University of Southern Denmark, Odense, Denmark; 4 Research Unit of Child and Adolescent Psychiatry, Mental Health Services in the Region of Southern Denmark, Odense, Denmark

**Keywords:** Postpartum psychiatry, Mediation analysis, Socioeconomic status, Body mass index, Personal psychiatric history

## Abstract

**Purpose:**

Socioeconomic status (SES) and body mass index (BMI) are risk factors for postpartum psychiatric episodes (PPE), but their relative contributions remain unclear. We examined how SES and BMI jointly influence PPE risk and whether BMI mediates the SES-PPE association across strata of psychiatric history.

**Methods:**

We conducted a register-based cohort study of 995,513 childbirths (2004-2021). SES was defined by an index of income, education, and employment, and pre-pregnancy BMI was grouped according to WHO criteria. PPE was defined as a psychiatric diagnosis or two redeemed prescriptions for psychotropic medications within 12 months postpartum. Psychiatric history was stratified as none, past, or recent. Logistic regression estimated associations between SES, BMI, and PPE by psychiatric history. Causal mediation analysis assessed BMI’s mediational role in the SES-PPE association stratified by psychiatric history, and population attributable risk percentage (PARP) quantified the proportion of cases attributable to SES and BMI.

**Results:**

Lower SES and BMI outside the normal range were independently associated with increased PPE risk, with effect sizes varying by psychiatric history. BMI mediated 1.26%-4.99% of the SES-PPE association, except for women with recent psychiatric history (21.37%). PARPs showed that SES accounted for a larger fraction of PPE cases (up to 31.40%) than BMI (4.48%-7.72%).

**Conclusion:**

Lower SES and BMI outside the normal ranges increase PPE risk, but SES has a stronger population-level impact than BMI. The SES-PPE association is largely independent of BMI, indicating that factors related to social disadvantage – not BMI – drive most of the excess risk.

**Supplementary Information:**

The online version contains supplementary material available at https://doi.org/10.1007/s00127-026-03089-1.

## Introduction

Postpartum psychiatric episodes (PPE) range from the rare but severe postpartum psychosis (1-2 per 1,000 women) to the more common postpartum depression (PPD) and anxiety, affecting around 17% and 15% of mothers worldwide [1–3]. These disorders have profound consequences, including increased maternal morbidity and mortality, impaired child development, heightened strain on family dynamics, and substantial economic costs [4–6]. Multiple risk factors contribute to PPE [7–10], including psychological vulnerabilities such as personal, family, and partner psychiatric history [11–13], hormonal sensitivities [14], pregnancy and birth complications [15–19], social determinants like socioeconomic status (SES) [20], adverse life events [21, 22], limited support [23, 24], and biological factors including genetic predisposition [25, 26] and body mass index (BMI) [27]. Collectively, these domains illustrate the complexity of PPE etiology, which may contribute to their frequent underdiagnosis and highlight the importance of elucidating underlying mechanisms [28, 29].

Evidence from the general population shows clear associations between BMI and mental health, particularly among women [30–32]. A meta-analysis of Mendelian randomization studies linked obesity to increased risk of depression, while a cohort study of adolescents reported a U-shaped association between BMI and mental health that strengthened over time [30, 31]. Consistent with this, pre-pregnancy underweight and overweight/obesity have been associated with elevated risk of PPD and, to a lesser extent, anxiety [27, 33]. Proposed mechanisms for this association include biological and social processes [34]. Importantly, BMI follows a clear social gradient: higher BMI is more prevalent among women from lower SES, potentially explaining part of the socioeconomic disparities in maternal mental health outcomes [35, 36].

Psychiatric history is the most consistently identified PPE risk factor, reflecting psychiatric vulnerabilities accumulated across the life course [12, 37]. Women with prior depression, anxiety, or other psychiatric disorders face a substantially elevated risk of PPE, making it essential to account for psychiatric history when assessing postpartum risk and failing to do this will result in biased estimates. As with BMI, psychiatric history is unequally distributed across social groups, with lower SES increasing the likelihood of prior psychiatric morbidity [38, 39].

Many PPE risk factors are deeply interconnected, yet they are often analyzed in isolation [14]. This interconnectedness is evident in the case of SES, as it shapes both physical health (e.g., BMI) and psychological vulnerability (e.g., psychiatric history) [35, 36, 38, 39]. Although SES is one of the strongest predictors of mental health outcomes in the general population, its role in PPE has received relatively little attention [20, 21]. Understanding how SES interacts with and amplifies other risk factors is critical for clarifying mechanisms underlying PPE.

The present study aimed to examine how SES and pre-pregnancy BMI jointly influence the risk of PPE, while accounting for the mother’s psychiatric history. Specifically, we sought to: (1) examine the independent associations of SES and PPE, and BMI and PPE, stratified by psychiatric history; (2) examine whether the association between SES and PPE is partly mediated by BMI, and whether this pathway differs according to psychiatric history; and (3) quantify the proportion of PPE cases attributable to SES and BMI to assess their population-level impact.

Conducting this study, we hypothesized that both SES and BMI would be independent risk factors for PPE, with BMI exerting a more modest effect, as it partly reflects underlying social determinants.

## Methods

This study was reported according to the AGReMA guideline, and the pre-registered protocol is available (https://osf.io/psqk4/) [40].

### Study design, participants, and follow-up

This nationwide register-based cohort study covered the period from January 1, 2004, to December 31, 2021. The Danish Civil Registration System, which includes all individuals residing in Denmark since 1968, enabled linkage of information across national health and administrative registries [41, 42]. The study population was restricted to women who gave birth to a live neonate at any gestation during the study period and for whom information on both SES and pre-pregnancy BMI was available. Stillbirths were excluded to maintain focus on PPE following a live birth, as the loss of a child entails distinct psychological consequences. BMI values below 12 or above 50 were excluded, as they were considered unrealistic and likely due to data entry errors.

### Outcome

The outcome, PPE, was defined as either a registered psychiatric in- or outpatient diagnosis – excluding substance use disorders and intellectual disabilities – (International Classification of Diseases, 10^th^ Revision (ICD-10): F00-99, excluding F10-19, F70-79), or two redeemed prescriptions for psychotropic medication used to treat mental health conditions (Anatomical Therapeutic Chemical (ATC): N05-06) within 12 months postpartum. Substance use disorders were excluded because they represent a distinct category of psychiatric disorders with different etiology and treatment pathways compared with other psychiatric disorders. The variable was categorized as *yes* or *no*. Diagnostic information was obtained from the Danish National Patient Register and the Danish Psychiatric Central Research Register, while prescription data were obtained from the Danish National Prescription Register [43–45].

### Exposure

The primary exposure, SES, was defined using an index in which the mother received one point for each of the following criteria, if present: (a) gross income in the lowest quintile, (b) being unemployed or outside the labor market, and (c) below high school level of education. This resulted in a four-level ordered variable ranging from 0 to 3, which we labelled as *high SES*, *medium-high SES*, *medium-low SES*, and *low SES* [46]. SES indicators were measured in the calendar year preceding the pregnancy (as of December 31). Information on income and employment was obtained from the Employment Classification Module and the Integrated Database for Labour Market Research, while education data were obtained from the Population Education Register [47, 48].

### Mediator

The mediator, BMI, was defined as weight in kilograms per square meter (kg/m^2^). Pre-pregnancy BMI data were obtained from the Medical Birth Register and categorized according to the World Health Organization (WHO) criteria: *underweight* (<18.5), *normal weight* (18.5-24.9), *pre-obesity* (25.0-29.9), *obesity class I* (30.0-34.9), and *obesity class II/III* (≥35.0) [49, 50]. BMI was measured approximately 7-9 weeks after conception during the first visit to the general practitioner to confirm pregnancy.

### Effect modifier

Psychiatric history was included as an effect modifier to examine whether the associations of SES and BMI with PPE differed between women with and without prior psychiatric vulnerability [51–53]. Women were classified as having a psychiatric history if they had any hospital-based diagnoses (ICD-8: 290-310, excluding 291.x9, 294.39, 303.xx, 304.xx; ICD-10: F00-99, excluding F10-19, F70-79) recorded in the Danish National Patient Register or the Danish Psychiatric Central Research Register, or if they redeemed at least two prescriptions for psychotropic medications (ATC: N05-06) recorded in the Danish National Prescription Register prior to conception. Psychiatric history was further categorized by timing: *recent* (≤ 2 years prior to conception) or *past* (>2 years prior to conception) and were mutually exclusive; women with both past and recent psychiatric history were classified as having recent psychiatric history. See Supplement (eFigure 1) for further details on variables and temporal order.

### Confounders

Potential confounders included year of delivery, maternal age at delivery, parity, and family psychiatric history, as these factors may influence the associations between SES, BMI, and PPE. Year of delivery and maternal age at delivery were obtained from the Civil Registration System, and maternal age was divided into quintiles [41]. Parity was obtained from the Medical Birth Register and categorized as *1*, *2*, and *3+* [50]. Family psychiatric history was defined as at least one parent of the index mother having received either a psychiatric diagnosis (ICD-8: 290-310, excluding 291.x9, 294.39, 303.xx, 304.xx; ICD-10: F00-99, excluding F10-19, F70-79) or having redeemed at least two prescriptions for psychotropic medication (ATC: N05-06) prior to delivery [43–45]. Smoking and obstetric complications were not included as confounders, as they were expected to be part of the causal pathway.

### Statistical analysis

Counts and frequencies were used to summarize characteristics of the study population, stratified by the exposure, SES.

The analysis consisted of three steps. In the first step, we estimated the independent associations between SES and PPE and between BMI and PPE, stratified by psychiatric history. Odds ratios (ORs) with 95% confidence intervals (CIs) were calculated using logistic regression. All models were adjusted for year of delivery, maternal age at delivery, parity, and family psychiatric history. To account for non-independence of multiple births contributed by the same women across different pregnancies, cluster robust standard errors, clustered by the mother’s personal identifier, were applied.

In the second step, we evaluated the potential mediating role of pre-pregnancy BMI in the association between SES and PPE using causal mediation analysis under a counterfactual framework [54, 55]. This approach decomposed the total effect of SES on PPE into natural direct and indirect pathways through BMI. The natural direct effect (NDE) represents the effect of SES on PPE, independent of BMI, while the natural indirect effect (NIE) captures the proportion of the effect mediated through BMI. The proportion mediated was calculated as the ratio of the NIE to the total effect (NIE+NDE), representing the percentage of the total effect explained by BMI. Mediation analyses were stratified by psychiatric history to assess whether associations differed between women with and without a psychiatric history. Estimates were generated with the R package *mediation* (version 4.5.0), with models adjusted for the same set of confounders as in the logistic regression [55]. Cluster robust standard errors were again applied to account for correlated data from repeated pregnancies, and 95% CI were obtained using a quasi-Bayesian approximation with 100 resamples.

In the third step, we assessed the population-level impact of SES and BMI on PPE by calculating the population attributable risk percentage (PARP). The PARP represents the proportion of PPE cases in the population that could be prevented if the exposure (low SES or BMI outside the normal range) were eliminated, assuming causal associations. PARP estimates were derived from adjusted logistic regression models, incorporating the prevalence of exposure in the study population together with the corresponding adjusted ORs.

Sensitivity analyses were performed. First, the main analyses were restricted to primiparous women to address dependency between observations. Second, the e-value was estimated to quantify the extent of unmeasured confounding required to explain away the point estimates and the lower CIs of the NIE and the NDE [56].

All analyses and plots were generated using R version 4.4.1.

### Ethics

The study was approved by the Danish Data Protection Agency (through local registration at Aarhus University, journal number 2016-051-000001, serial number 2304). All data were anonymized and analyzed on a secure platform at Statistics Denmark. Informed consent was not required in accordance with Danish law.

## Results

During the study period (January 1, 2004, to December 31, 2021), there were 1,119,869 childbirths in Denmark, of which 11,591 (1.0%) were stillbirths and thus excluded. An additional 54,026 (4.8%) births were excluded due to missing or unrealistic maternal BMI values, and 58,739 (5.2%) were excluded due to missing information on one of the components of the SES index (income, educational attainment, or labor market status). This resulted in a final study population of 995,513 (88.9%) mothers who gave birth to a live child (Supplement, eFigure 2).

Table 1 presents the baseline characteristics of the study population, stratified by SES. Of the study population, 621,931 (62.5%) were in the high SES group, 207,475 (20.8%) in the medium-high SES group, 140,500 (14.1%) in the medium-low SES group, and 25,607 (2.6%) in the low SES group. Mothers in the low SES group more frequently experienced PPE compared with those in the high SES group (10.5% vs. 4.2%). They were also more likely to have a BMI ≥35 (7.6% vs. 3.8%), have a psychiatric history (35.3%. vs. 17.6%), were younger (<26.9 years: 36.1% vs. 9.6%), and were more often first-time mothers (52.0% vs. 43.3%). A slightly higher proportion of mothers with a family psychiatric history belonged to the high SES group compared with the low SES group (52.0% vs. 48.9%).

**Table 1:** Characteristics of the study population

Characteristic	SES index			
	High, N = 621,931 (62.5%)	Medium-high, N = 207,475 (20.8%)	Medium-low, N = 140,500 (14.1%)	Low, N = 25,607 (2.6%)
Postpartum psychiatric episodes (PPE)				
No	595,908 (95.8%)	193,254 (93.1%)	125,813 (89.5%)	22,912 (89.5%)
Yes	26,023 (4.2%)	14,221 (6.9%)	14,687 (10.5%)	2,695 (10.5%)
Body Mass Index (BMI)				
Underweight	20,513 (3.3%)	9,147 (4.4%)	8,761 (6.2%)	1,687 (6.6%)
Normal weight	394,372 (63.4%)	122,770 (59.2%)	79,056 (56.3%)	12,635 (49.3%)
Pre-obesity	134,036 (21.6%)	45,642 (22.0%)	30,473 (21.7%)	6,269 (24.5%)
Obesity class I	49,236 (7.9%)	19,425 (9.4%)	13,871 (9.9%)	3,065 (12.0%)
Obesity class II/III	23,774 (3.8%)	10,491 (5.1%)	8,339 (5.9%)	1,951 (7.6%)
Psychiatric history				
None	512,543 (82.4%)	155,754 (75.1%)	93,988 (66.9%)	16,563 (64.7%)
Past	74,172 (11.9%)	30,154 (14.5%)	22,588 (16.1%)	4,377 (17.1%)
Recent	35,216 (5.7%)	21,567 (10.4%)	23,924 (17.0%)	4,667 (18.2%)
Familial psychiatric history				
No	298,286 (48.0%)	94,225 (45.4%)	64,288 (45.8%)	13,097 (51.1%)
Yes	323,645 (52.0%)	113,250 (54.6%)	76,212 (54.2%)	12,510 (48.9%)
Age at delivery				
<26.9 years	59,921 (9.6%)	76,611 (36.9%)	53,313 (37.9%)	9,243 (36.1%)
26.9-29.5 years	118,493 (19.1%)	52,658 (25.4%)	25,017 (17.8%)	2,819 (11.0%)
29.6-32.0 years	143,645 (23.1%)	30,418 (14.7%)	21,830 (15.5%)	3,222 (12.6%)
32.1-35.0 years	151,943 (24.4%)	23,663 (11.4%)	19,400 (13.8%)	4,183 (16.3%)
≥35.1 years	147,929 (23.8%)	24,125 (11.6%)	20,940 (14.9%)	6,140 (24.0%)
Year of delivery				
2004-2009	205,157 (33.0%)	71,700 (34.6%)	53,568 (38.1%)	9,940 (38.8%)
2010-2015	201,113 (32.3%)	65,392 (31.5%)	43,823 (31.2%)	7,922 (30.9%)
2016-2021	215,661 (34.7%)	70,383 (33.9%)	43,109 (30.7%)	7,745 (30.2%)
Parity				
1	268,997 (43.3%)	107,610 (51.9%)	62,580 (44.5%)	13,321 (52.0%)
2	255,876 (41.1%)	71,270 (34.4%)	51,010 (36.3%)	7,097 (27.7%)
3+	97,058 (15.6%)	28,595 (13.8%)	26,910 (19.2%)	5,189 (20.3%)

**Fig. 1:**
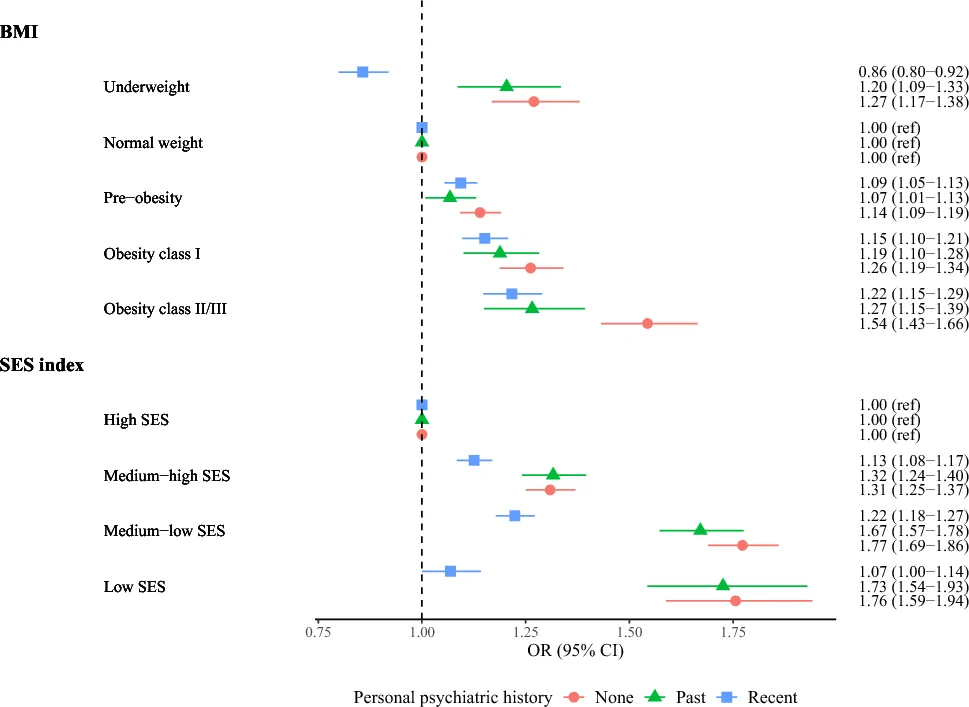
Associations between BMI and PPE, as well as SES and PPE, stratified by personal psychiatric history

Figure 1 shows the associations between BMI and PPE, as well as SES and PPE, stratified by personal psychiatric history. For BMI, a gradient was observed, with higher BMI categories associated with increasing risk of PPE. In the pre-obesity category, the risk of PPE was elevated compared with the normal weight group, but the magnitude of the increase did not differ substantially across psychiatric history within the pre-obesity group (none: OR=1.14, [95% CI=1.09;1.19]; past: OR=1.07, [95% CI=1.01;1.13]; recent: OR=1.09, [95% CI=1.05;1.13]). Similarly, in obesity class I, risk estimates were increased compared with the normal weight group, but the risk was increased similarly across psychiatric history within the obesity class I category (none: OR=1.26, [95% CI=1.19;1.34]; past: OR=1.19, [95% CI=1.10;1.28]; recent: OR=1.15, [95% CI=1.10;1.21]). In obesity class II, women without a psychiatric history had the highest relative risk of PPE (OR=1.54, [95% CI=1.43;1.66]), whereas those with past (OR=1.27, [95% CI=1.15;1.39]) or recent (OR=1.22, [95% CI=1.15;1.29]) psychiatric history had lower but similar risk estimates. Among underweight women, the risk of PPE was significantly higher for those with no (OR=1.27, [95% CI=1.17;1.38]) or past (OR=1.20, [95% CI=1.09;1.33]) psychiatric history, while women with a recent psychiatric history had a lower risk compared with the reference group (OR=0.86, [95% CI=0.80;0.92]). For SES, compared with women in the high SES group, increased risk of PPE was observed among women in the medium-high, medium-low, and low SES groups. The relative risk was highest in the medium-low (none: OR=1.77, [95% CI=1.69;1.86]; past: OR=1.67, [95% CI=1.57;1.78]) and low (none: OR=1.76, [95% CI=1.59;1.94]; past: OR=1.73, [95% CI=1.54;1.93]) SES groups for women with no or past psychiatric history. In contrast, the risk was less increased among women with a recent psychiatric history compared to the high SES group (medium-low SES: OR=1.22, [95% CI=1.18;1.27]; low SES: OR=1.07, [95% CI=1.00;1.14]). Although point estimates suggested gradients across BMI and SES categories, CIs largely overlapped, indicating limited evidence for statistically distinct differences between adjacent categories.

Table 2 present the results of the mediation analysis examining the role of BMI in the association between SES and PPE, stratified by psychiatric history. Overall, the proportion mediated by BMI was low. For the medium-high and medium-low SES groups, the proportion mediated was 1.85% and 1.26% among women with no psychiatric history, 1.56% and 1.56% among women with past psychiatric history, and 4.99% and 4.60% among women with recent psychiatric history. For the low SES group, the proportion mediated was slightly higher, at 4.56%, 3.01%, and 21.37% for women with no, past, or recent psychiatric history, respectively.

**Table 2:** Mediation analysis of association between SES and PPE with BMI as mediator, stratified by psychiatric history

SES index	Direct effect	Indirect effect	Proportion mediated
**No psychiatric history**			
High → Medium-high	1.004 (1.004,1.005)	1.000 (1.000,1.000)	1.85%
High → Medium-low	1.011 (1.010,1.012)	1.000 (1.000,1.000)	1.26%
High → Low	1.011 (1.008,1.013)	1.001 (1.000,1.001)	4.56%
**Past psychiatric history**			
High → Medium-high	1.016 (1.012,1.020)	1.000 (1.000,1.000)	1.56%
High → Medium-low	1.033 (1.029,1.038)	1.001 (1.000,1.001)	1.56%
High → Low	1.036 (1.026,1.046)	1.001 (1.001,1.002)	3.01%
**Recent psychiatric history**			
High → Medium-high	1.028 (1.017,1.039)	1.001 (1.001,1.002)	4.99%
High → Medium-low	1.048 (1.037,1.057)	1.002 (1.002,1.003)	4.60%
High → Low	1.013 (0.996,1.031)	1.004 (1.002,1.005)	21.37%

Table 3 presents the PARP for BMI and SES in relation to PPE, stratified by psychiatric history. For BMI, the PARP was 7.72% among women with no psychiatric history, 5.38% among women with past psychiatric history, and 4.48% among women with recent psychiatric history. For SES, the PARP was 23.81%, 31.40%, and 10.09% among women with no, past, or recent psychiatric history.

**Table 3:** Population attributable risk percentage (PARP) for BMI and SES, stratified on psychiatric history

	Psychiatric history		
	None	Past	Recent
BMI	7.72%	5.38%	4.48%
SES	23.81%	31.40%	10.09%

### Sensitivity analyses

Restricting the study population to primiparous women yielded results consistent with those observed in the main analyses (eTables 1-3). The e-value, which quantifies the strength of unmeasured confounding required to fully explain the observed mediational effect, indicated that only a small degree of unmeasured confounding would be sufficient, consistent with the finding of little to no mediation (eTable 4).

## Discussion

### Main findings

In this cohort study of 995,513 childbirths in Denmark, we found that both BMIs outside the normal weight range and lower SES were associated with increased PPE risk, although the strength of these associations differed according to the mothers’ prior psychiatric history. Mediation analyses indicated that BMI explained only a small proportion of the association between SES and PPE, while population attributable risk estimates suggested that SES contributes substantially more to the overall burden of PPE than BMI.

### Interpretation and current evidence

Our findings align with existing evidence demonstrating a clear social gradient: mothers in lower SES groups had higher prevalence of psychiatric history, obesity, and were younger at childbirth [35, 36, 38, 39]. These disparities highlight the clustering of risk factors among socially disadvantaged women and underline the importance of considering SES in studies of PPE.

When examining the associations between BMI and PPE stratified by psychiatric history, a clear pattern emerged. Across all BMI categories above normal weight, the risk of PPE was increased compared with the normal weight group. Within each BMI category, mothers with no psychiatric history generally had the highest risk. This pattern is likely explained by the fact that women with a psychiatric history have a substantially higher baseline risk of PPE than women without a psychiatric history, as prior psychiatric disorders, especially recent episodes, are the strongest PPE risk factor [12, 57]. As a result, the observable relative contribution of BMI is more limited for women with past, and especially, recent, psychiatric history. Another important consideration is that psychiatric history was defined based on psychiatric diagnoses or psychotropic medication use prior to conception. Women classified as having a recent psychiatric history therefore represent a group with identified psychiatric conditions and are likely to receive ongoing clinical follow-up before, during, and after pregnancy. Such continuity of care, together with their substantially higher baseline risk of PPE, may reduce the observable relative contribution of additional risk factors and could partly explain the attenuated associations observed in this group. A similar, though, not identical pattern was observed among underweight women, where risk was elevated for those with no or past psychiatric history, while women with a recent psychiatric history had lower risk. Similarly, the associations between SES and PPE revealed a social gradient. Women in lower SES groups consistently exhibited higher risk of PPE compared with women in the high SES group. These findings should be interpreted in the context of age-related accumulation of education, income, and labour market attachment, as SES tends to increase over the life course. The gradient was most pronounced among women with no or past psychiatric history, whereas women with a recent psychiatric history showed a smaller increase in risk across SES groups. This pattern is consistent with the notion that, for women with recent psychiatric history, baseline risk is already very high, which may attenuate the relative effect of SES [12]. Together, while research on the relationship between SES and PPE is relatively limited, our findings are in line with studies suggesting that lower SES is associated with increased risk of PPE, as well as studies showing clear associations between BMI and PPE [20, 21, 27, 33]. However, their relative contributions differ depending on prior psychiatric vulnerability, highlighting the importance of considering interactions between maternal characteristics and psychiatric history. While consistent gradients across BMI and SES categories were observed, the overlap of CIs suggests that differences between specific categories should be interpreted with caution.

The mediation analysis indicated that BMI explains only a small proportion of the association between SES and PPE, regardless of psychiatric history, suggesting that most of the increased risk associated with lower SES is driven by other mechanisms. Our findings are consistent with SES acting as a common underlying determinant of both BMI outside the normal range and PPE. The limited mediating role of BMI also suggests that low SES likely shapes vulnerability through multiple pathways. In the Danish population, low SES has been associated with lower utilization and poorer quality of mental healthcare, while in broader international evidence, it is linked to greater exposure to stress, adverse life events, and limited social resources [58–61]. These overlapping disadvantages may overshadow the role of BMI, even though obesity is more common in socially disadvantaged groups. Thus, SES should be understood not as a proxy for lifestyle factors alone, but as a broader determinant of maternal mental health operating through social, psychological, and structural mechanisms.

The PARP highlights the relative contributions of BMI and SES to the overall population-level burden of PPE. For BMI, the PARP was modest across all strata of maternal psychiatric history, ranging from 4.48% to 7.72%, indicating only a small fraction of PPE cases in the population could be attributed to elevated or low BMI. In contrast, SES accounted for a substantially larger proportion of PPE, particularly among women with no or past psychiatric history, with PARPs of 23.81% and 31.40%. Among women with recent psychiatric history, the PARP for SES was lower (10.09%), reflecting that the high baseline risk in this group limits the observable relative contribution of additional risk factors. Overall, these findings suggest that, while BMI is a detectable risk factor for PPE, social disadvantage represents a more substantial population-level determinant of PPE risk. The differential PARPs across psychiatric history strata further emphasizes that the impact of SES and BMI on PPE varies depending on prior maternal vulnerability.

### Strengths and limitations

First, this study is population-based and includes over 995,000 childbirths, allowing for examination of subgroups stratified by psychiatric history, an important determinant of PPE risk. Second, the large sample size enables stratification instead of applying a washout period, allowing us to retain women with prior psychiatric disorders and thereby capture a vulnerable population often excluded in other studies. Third, comprehensive linkage across Danish national registers provides detailed information on health and social factors, including BMI, SES measures (income, labour market status, and educational attainment), as well as psychiatric diagnoses and medication use, which is rarely available on this scale.

However, there are also limitations. First, women experiencing stillbirths or spontaneous abortions were excluded. Previous studies suggest a social gradient in stillbirth even in high-income countries, so excluding these women may alter the composition of the study population and potentially underestimate risks in socially disadvantaged groups [62, 63]. Second, we did not apply a washout period prior to birth to differentiate pre- and post-birth psychiatric illnesses. Instead, we relied on stratification by prior psychiatric diagnoses, which, while retaining vulnerable women in the analysis, may not perfectly distinguish the timing of onset. Third, some misclassification is possible if women received a postpartum diagnosis that had onset prior to delivery, which could slightly bias the observed associations. Fourth, family psychiatric history may be under-ascertained due to left truncation, as psychiatric diagnoses are only available from 1968 and prescription data from 1995. Psychiatric morbidity occurring before the establishment of these registers is therefore not captured, particularly for parents born in earlier cohorts. Finally, missing data on BMI (4.8%) and SES (5.2%) resulted in the exclusion of 10%. Complete-case analysis was therefore used. This approach relies on the assumption that excluded individuals are comparable to those included with respect to the associations under study, which may not fully hold. Missing information on SES and BMI is likely more common among socioeconomically disadvantaged women and those with limited contact with healthcare services, leading to potential underrepresentation of the most vulnerable groups and thereby underestimating the associations.

## Conclusion

In this large, population-based cohort of over 995,000 Danish childbirths, low SES contributed substantially to the population-level burden of PPE, likely through multiple social, psychological, and structural pathways beyond maternal BMI. Both lower SES and pre-pregnancy BMI outside the normal range were associated with increased risk of PPE, with associations varying by prior psychiatric history – the strongest single risk factor for PPE and therefore an essential dimension to account for. BMI explained only a small fraction of the SES-PPE association. These findings highlight the importance of considering socioeconomic disadvantage alongside psychiatric history in identifying women at highest risk of PPE. While SES itself cannot be modified, it captures a wide range of disadvantage, underscoring the need to ensure sufficient and tailored support for vulnerable women, many of whom face challenges across multiple domains.

## Supplementary Information

Below is the link to the electronic supplementary material.

Supplementary file1 (PDF 198 KB)

## Data Availability

Data used in this study are derived from healthcare records and Danish national registers and contain sensitive personal information. In accordance with Danish data protection regulations, individual-level data cannot be shared publicly. Access may be granted to researchers who meet the criteria for access to confidential data through application to Statistics Denmark.

## References

[1] Sit D, Rothschild AJ, Wisner KL (2006) A review of postpartum psychosis. J Womens Health 15:352–368. 10.1089/jwh.2006.15.352 PMC3109493 16724884

[2] Wang Z, Liu J, Shuai H, Cai Z, Fu X, Liu Y et al (2021) Mapping global prevalence of depression among postpartum women. Transl Psychiatry 11:543. 10.1038/s41398-021-01663-6 34671011 PMC8528847

[3] Dennis C-L, Falah-Hassani K, Shiri R (2017) Prevalence of antenatal and postnatal anxiety: systematic review and meta-analysis. Br J Psychiatry 210:315–323. 10.1192/bjp.bp.116.187179 28302701

[4] Slomian J, Honvo G, Emonts P, Reginster J-Y, Bruyère O (2019) Consequences of maternal postpartum depression: a systematic review of maternal and infant outcomes. Womens Health 15:1745506519844044. 10.1177/1745506519844044 PMC6492376 31035856

[5] Johannsen BMW, Mægbæk ML, Bech BH, Laursen TM, Munk-Olsen T (2021) Divorce or separation following postpartum psychiatric episodes: a population-based cohort study. J Clin Psychiatry 82:20m13555. 10.4088/JCP.20m13555 34033272

[6] Bauer A, Knapp M, Parsonage M (2016) Lifetime costs of perinatal anxiety and depression. J Affect Disord 192:83–90. 10.1016/j.jad.2015.12.005 26707352

[7] Gastaldon C, Solmi M, Correll CU, Barbui C, Schoretsanitis G (2022) Risk factors of postpartum depression and depressive symptoms: umbrella review of current evidence from systematic reviews and meta-analyses of observational studies. Br J Psychiatry 221:591–602. 10.1192/bjp.2021.222 35081993

[8] Hutchens BF, Kearney J (2020) Risk factors for postpartum depression: an umbrella review. J Midwifery Womens Health 65:96–108. 10.1111/jmwh.13067 31970924

[9] Kim JH, Kim JY, Lee S, Lee S, Stubbs B, Koyanagi A et al (2022) Environmental risk factors, protective factors, and biomarkers for postpartum depressive symptoms: an umbrella review. Neurosci Biobehav Rev 140:104761. 10.1016/j.neubiorev.2022.104761 35803397

[10] Zhao XH, Zhang ZH (2020) Risk factors for postpartum depression: an evidence-based systematic review of systematic reviews and meta-analyses. Asian J Psychiatry 53:102353. 10.1016/j.ajp.2020.102353 32927309

[11] Zacher Kjeldsen M-M, Bricca A, Liu X, Frokjaer VG, Madsen KB, Munk-Olsen T (2022) Family history of psychiatric disorders as a risk factor for maternal postpartum depression: a systematic review and meta-analysis. JAMA Psychiat 79:1004–1013. 10.1001/jamapsychiatry.2022.2400 PMC9386615 35976654

[12] Zacher Kjeldsen M-M, Bang Madsen K, Liu X, Mægbæg ML, Robakis T, Bergink V et al (2024) Identifying postpartum depression: using key risk factors for early detection. BMJ Ment Health 27:e301206. 10.1136/bmjment-2024-301206 39353685 PMC11448151

[13] Zacher Kjeldsen M-M, Liu X, Bang Madsen K, Elliott AF, Zacher Kjeldsen S, Mægbæk ML et al (2025) Partner status and partner mental health and the risk of postpartum depression. Nat Ment Health 3:814–820. 10.1038/s44220-025-00461-z

[14] Yim IS, Tanner Stapleton LR, Guardino CM, Hahn-Holbrook J, Schetter CD (2015) Biological and psychosocial predictors of postpartum depression: systematic review and call for integration. Annu Rev Clin Psychol 11:99–137. 10.1146/annurev-clinpsy-101414-020426 25822344 PMC5659274

[15] Caropreso L, de Azevedo Cardoso T, Eltayebani M, Frey BN (2020) Preeclampsia as a risk factor for postpartum depression and psychosis: a systematic review and meta-analysis. Arch Womens Ment Health 23:493–505. 10.1007/s00737-019-01010-1 31802249

[16] Kang SY, Kim H-B, Sunwoo S (2020) Association between anemia and maternal depression: a systematic review and meta-analysis. J Psychiatr Res 122:88–96. 10.1016/j.jpsychires.2020.01.001 31945502

[17] Schoretsanitis G, Gastaldon C, Ochsenbein‐Koelble N, Olbrich S, Barbui C, Seifritz E (2024) Postpartum hemorrhage and postpartum depression: a systematic review and meta‐analysis of observational studies. Acta Psychiatr Scand 150:274–283. 10.1111/acps.13583 37286177

[18] Sun L, Wang S, Li X-Q (2021) Association between mode of delivery and postpartum depression: a systematic review and network meta-analysis. Aust N Z J Psychiatry 55:588–601. 10.1177/0004867420954284 32929976

[19] de Paula Eduardo JAF, de Rezende MG, Menezes PR, Del-Ben CM (2019) Preterm birth as a risk factor for postpartum depression: a systematic review and meta-analysis. J Affect Disord 259:392–403. 10.1016/j.jad.2019.08.069 31470184

[20] Szurek-Cabanas R, Navarro-Carrillo G, Martínez-Sánchez CA, Oyanedel JC, Villalobos D (2024) Socioeconomic status and maternal postpartum depression: a PRISMA-compliant systematic review. Curr Psychol 43:27339–27350. 10.1007/s12144-024-05774-3

[21] Guintivano J, Manuck T, Meltzer-Brody S (2018) Predictors of postpartum depression: a comprehensive review of the last decade of evidence. Clin Obstet Gynecol 61:591–603. 10.1097/GRF.0000000000000368 29596076 PMC6059965

[22] Meltzer-Brody S, Larsen JT, Petersen L, Guintivano J, Florio AD, Miller WC et al (2018) Adverse life events increase risk for postpartum psychiatric episodes: a population-based epidemiologic study. Depress Anxiety 35:160–167. 10.1002/da.22697 29172228 PMC6867605

[23] Khademi K, Kaveh MH (2024) Social support as a coping resource for psychosocial conditions in postpartum period: a systematic review and logic framework. BMC Psychol 12:301. 10.1186/s40359-024-01814-6 38807228 PMC11131291

[24] Bedaso A, Adams J, Peng W, Sibbritt D (2021) The relationship between social support and mental health problems during pregnancy: a systematic review and meta-analysis. Reprod Health 18:162. 10.1186/s12978-021-01209-5 34321040 PMC8320195

[25] Guintivano J, Byrne EM, Kiewa J, Yao S, Bauer AE, Aberg KA et al (2023) Meta-analyses of genome-wide association studies for postpartum depression. Am J Psychiatry 180:884–895. 10.1176/appi.ajp.20230053 37849304 PMC11163373

[26] Elwood J, Murray E, Bell A, Sinclair M, Kernohan WG, Stockdale J (2019) A systematic review investigating if genetic or epigenetic markers are associated with postnatal depression. J Affect Disord 253:51–62. 10.1016/j.jad.2019.04.059 31029013

[27] Dachew BA, Ayano G, Betts K, Alati R (2021) The impact of pre-pregnancy BMI on maternal depressive and anxiety symptoms during pregnancy and the postpartum period: a systematic review and meta-analysis. J Affect Disord 281:321–330. 10.1016/j.jad.2020.12.010 33341015

[28] Dagher RK, Bruckheim HE, Colpe LJ, Edwards E, White DB (2021) Perinatal depression: challenges and opportunities. J Womens Health Larchmt 30:154–159. 10.1089/jwh.2020.8862 33156730 PMC7891219

[29] Stewart DE, Vigod SN (2019) Postpartum depression: pathophysiology, treatment, and emerging therapeutics. Annu Rev Med 70:183–196. 10.1146/annurev-med-041217-011106 30691372

[30] Chen S, Zhang H, Gao M, Machado DB, Jin H, Scherer N et al (2024) Dose-dependent association between body mass index and mental health and changes over time. JAMA Psychiat 81:797–806. 10.1001/jamapsychiatry.2024.0921 PMC11097104 38748415

[31] Jokela M, Laakasuo M (2023) Obesity as a causal risk factor for depression: systematic review and meta-analysis of Mendelian randomization studies and implications for population mental health. J Psychiatr Res 163:86–92. 10.1016/j.jpsychires.2023.05.034 37207436

[32] Luppino FS, de Wit LM, Bouvy PF, Stijnen T, Cuijpers P, Penninx BWJH et al (2010) Overweight, obesity, and depression: a systematic review and meta-analysis of longitudinal studies. Arch Gen Psychiatry 67:220–229. 10.1001/archgenpsychiatry.2010.2 20194822

[33] Bliddal M, Pottegård A, Kirkegaard H, Olsen J, Jørgensen JS, Sørensen TI et al (2015) Mental disorders in motherhood according to prepregnancy BMI and pregnancy-related weight changes—a Danish cohort study. J Affect Disord 183:322–329. 10.1016/j.jad.2015.04.053 26047960

[34] Markowitz S, Friedman MA, Arent SM (2008) Understanding the relation between obesity and depression: causal mechanisms and implications for treatment. Clin Psychol Sci Pract 15:1–20

[35] Lundberg CE, Ryd M, Adiels M, Rosengren A, Björck L (2021) Social inequalities and trends in pre-pregnancy body mass index in Swedish women. Sci Rep 11:12056. 10.1038/s41598-021-91441-7 34103588 PMC8187407

[36] Newton S, Braithwaite D, Akinyemiju TF (2017) Socio-economic status over the life course and obesity: systematic review and meta-analysis. PLoS ONE 12:e0177151. 10.1371/journal.pone.0177151 28510579 PMC5433719

[37] Meltzer-Brody S, Howard LM, Bergink V, Vigod S, Jones I, Munk-Olsen T et al (2018) Postpartum psychiatric disorders. Nat Rev Dis Primers 4:18022. 10.1038/nrdp.2018.22 29695824

[38] Weye NO, Plana-Ripoll O, Baravelli CM, Agardh EE, Van Der Velde L, Kinge JM et al (2024) Educational differences in years lived with disability due to mental and substance use disorders: a cohort study using nationwide Norwegian and Danish registries. BMC Public Health 24:2576. 10.1186/s12889-024-20064-0 39304880 PMC11416009

[39] Sørensen CLB, Plana-Ripoll O, Bültmann U, Winding TN, Steen PB, Biering K (2025) Social inequality in mental disorder diagnoses and psychotropic medication use among 15-year-old adolescents in Denmark from 2002–2022. Soc Psychiatry Psychiatr Epidemiol. 10.1007/s00127-025-02943-y 40603721 PMC12855378

[40] Lee H, Cashin AG, Lamb SE, Hopewell S, Vansteelandt S, VanderWeele TJ et al (2021) A guideline for reporting mediation analyses of randomized trials and observational studies: the AGReMA statement. JAMA 326:1045. 10.1001/jama.2021.14075 34546296 PMC8974292

[41] Pedersen CB (2011) The Danish Civil Registration System. Scand J Public Health 39:22–25. 10.1177/1403494810387965 21775345

[42] Schmidt M, Schmidt SAJ, Adelborg K, Sundbøll J, Laugesen K, Ehrenstein V et al (2019) The Danish health care system and epidemiological research: from health care contacts to database records. Clin Epidemiol 11:563–591. 10.2147/CLEP.S179083 31372058 PMC6634267

[43] Schmidt M, Schmidt SAJ, Sandegaard JL, Ehrenstein V, Pedersen L, Sørensen HT (2015) The danish national patient registry: a review of content, data quality, and research potential. Clin Epidemiol 7:449–490. 10.2147/CLEP.S91125 26604824 PMC4655913

[44] Pottegård A, Schmidt SAJ, Wallach-Kildemoes H, Sørensen HT, Hallas J, Schmidt M (2017) Data resource profile: the danish national prescription registry. Int J Epidemiol 46:798–798f. 10.1093/ije/dyw213 27789670 PMC5837522

[45] Mors O, Perto GP, Mortensen PB (2011) The Danish Psychiatric Central Research Register. Scand J Public Health 39:54–57. 10.1177/1403494810395825 21775352

[46] Debost J-C, Larsen JT, Munk-Olsen T, Mortensen PB, Agerbo E, Petersen LV (2019) Childhood infections and schizophrenia: the impact of parental SES and mental illness, and childhood adversities. Brain Behav Immun 81:341–347. 10.1016/j.bbi.2019.06.031 31247291

[47] Petersson F, Baadsgaard M, Thygesen LC (2011) Danish registers on personal labour market affiliation. Scand J Public Health 39:95–98. 10.1177/1403494811408483 21775363

[48] Jensen VM, Rasmussen AW (2011) Danish education registers. Scand J Public Health 39:91–94. 10.1177/1403494810394715 21775362

[49] World Health Organization, editor (2000) Obesity: preventing and managing the global epidemic. Report of a WHO consultation. Geneva: World Health Organization. 11234459

[50] Bliddal M, Broe A, Pottegård A, Olsen J, Langhoff-Roos J (2018) The Danish Medical Birth Register. Eur J Epidemiol 33:27–36. 10.1007/s10654-018-0356-1 29349587

[51] Andreu-Bernabeu Á, González-Peñas J, Arango C, Díaz-Caneja CM (2023) Socioeconomic status and severe mental disorders: a bidirectional multivariable Mendelian randomisation study. BMJ Ment Health 26:e300821. 10.1136/bmjment-2023-300821 38007229 PMC10680010

[52] Xu Q, Li H, Zhu D (2024) Socioeconomic status, personality, and major mental disorders: a bidirectional Mendelian randomization study. Schizophrenia 10:49. 10.1038/s41537-024-00471-3 38678036 PMC11055884

[53] Corraini P, Olsen M, Pedersen L, Dekkers O, Vandenbroucke J (2017) Effect modification, interaction and mediation: an overview of theoretical insights for clinical investigators. Clin Epidemiol 9:331–338. 10.2147/CLEP.S129728 28652815 PMC5476432

[54] Lange T, Vansteelandt S, Bekaert M (2012) A simple unified approach for estimating natural direct and indirect effects. Am J Epidemiol 176:190–195. 10.1093/aje/kwr525 22781427

[55] Lange T, Hansen KW, Sørensen R, Galatius S (2017) Applied mediation analyses: a review and tutorial. Epidemiol Health 39:e2017035. 10.4178/epih.e2017035 29121709 PMC5723912

[56] Ding P, Vanderweele TJ (2016) Sharp sensitivity bounds for mediation under unmeasured mediator-outcome confounding. Biometrika 103:483–490. 10.1093/biomet/asw012 27279672 PMC4890130

[57] Munk-Olsen T, Ingstrup KG, Johannsen BM, Liu X (2020) Population-based assessment of the recurrence risk of postpartum mental disorders: will it happen again? JAMA Psychiat 77:213–214. 10.1001/jamapsychiatry.2019.3208 PMC6802053 31617870

[58] Verbeek T, Bockting CLH, Beijers C, Meijer JL, van Pampus MG, Burger H (2019) Low socioeconomic status increases effects of negative life events on antenatal anxiety and depression. Women Birth 32:e138–e143. 10.1016/j.wombi.2018.05.005 29887508

[59] Businelle MS, Mills BA, Chartier KG, Kendzor DE, Reingle JM, Shuval K (2014) Do stressful events account for the link between socioeconomic status and mental health? J Public Health 36:205–212. 10.1093/pubmed/fdt060 PMC4041099 23764393

[60] Knudsen SV, Valentin JB, Videbech P, Mainz J, Johnsen SP (2022) Inequities in mental health care quality and clinical outcomes among inpatients with depression within a tax-financed universal health care system. Clin Epidemiol 14:803–813. 10.2147/CLEP.S322392 35789690 PMC9250345

[61] Packness A, Wehberg S, Hastrup LH, Simonsen E, Søndergaard J, Waldorff FB (2021) Socioeconomic position and mental health care use before and after first redeemed antidepressant and time until subsequent contact to psychologist or psychiatrists: a nationwide Danish follow-up study. Soc Psychiatry Psychiatr Epidemiol 56:449–462. 10.1007/s00127-020-01908-7 32642803 PMC7904708

[62] Seaton SE, Field DJ, Draper ES, Manktelow BN, Smith GCS, Springett A et al (2012) Socioeconomic inequalities in the rate of stillbirths by cause: a population-based study. BMJ Open 2:e001100. 10.1136/bmjopen-2012-001100 22735165 PMC3383980

[63] Zeitlin J, Mortensen L, Prunet C, Macfarlane A, Hindori-Mohangoo AD, Gissler M et al (2016) Socioeconomic inequalities in stillbirth rates in Europe: measuring the gap using routine data from the Euro-Peristat Project. BMC Pregnancy Childbirth 16:15. 10.1186/s12884-016-0804-4 26809989 PMC4727282

