# Supplementary material for: The role of socioeconomic status and pre-pregnancy BMI in postpartum psychiatric episodes – a mediation study
Source: Soc Psychiatry Psychiatr Epidemiol. 2026 Apr 20;61(10):2071–80. doi: 10.1007/s00127-026-03089-1 (PMC13586007; doi:10.1007/s00127-026-03089-1)
Supplement: Supplementary file 1 [file 127_2026_3089_MOESM1_ESM.pdf]

Supplement

eFigure 1 Details of variables and temporal order

eFigure 2 Flowchart of study population

eTable 1 Odds ratios and 95% CIs for PPE restricted to primiparous women and stratified on psychiatric history

eTable 2 Mediation analysis with SES as exposure, BMI as mediator, and PPE as outcome restricted to primiparous women. Stratified on psychiatric history

eTable 3 Proportion attributable risk percentage (PARP) for BMI and SES on PPE, restricted to primiparous women. Stratified on psychiatric history

eTable 4 E-value for the indirect and direct effect

eFigure 1. Details of variables and temporal order

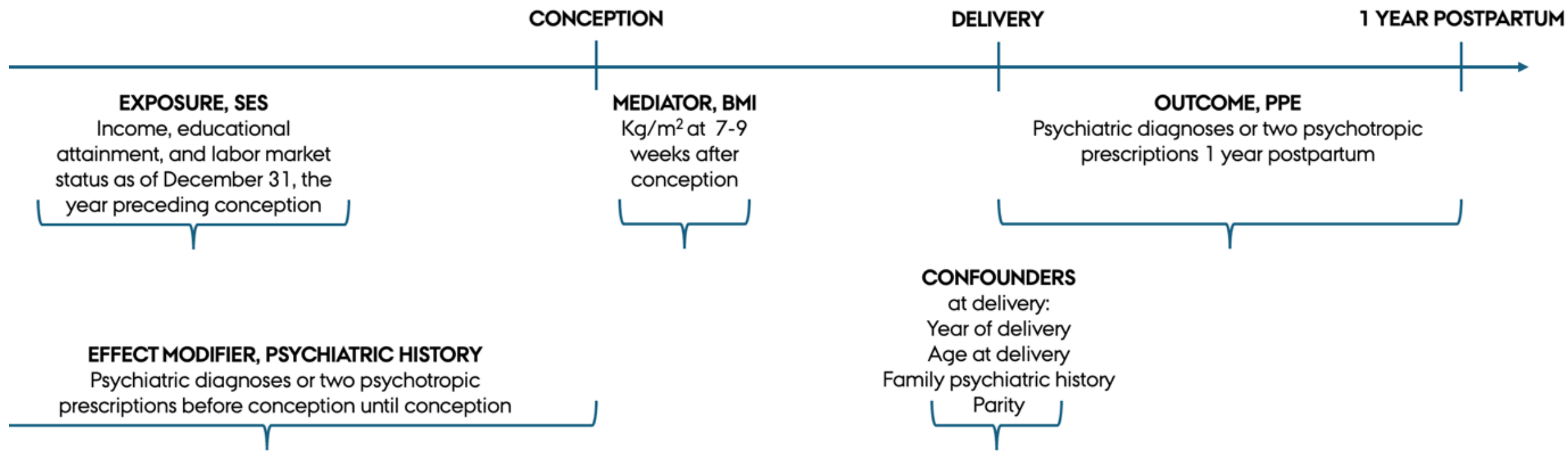

eFigure 2. Flowchart of study population

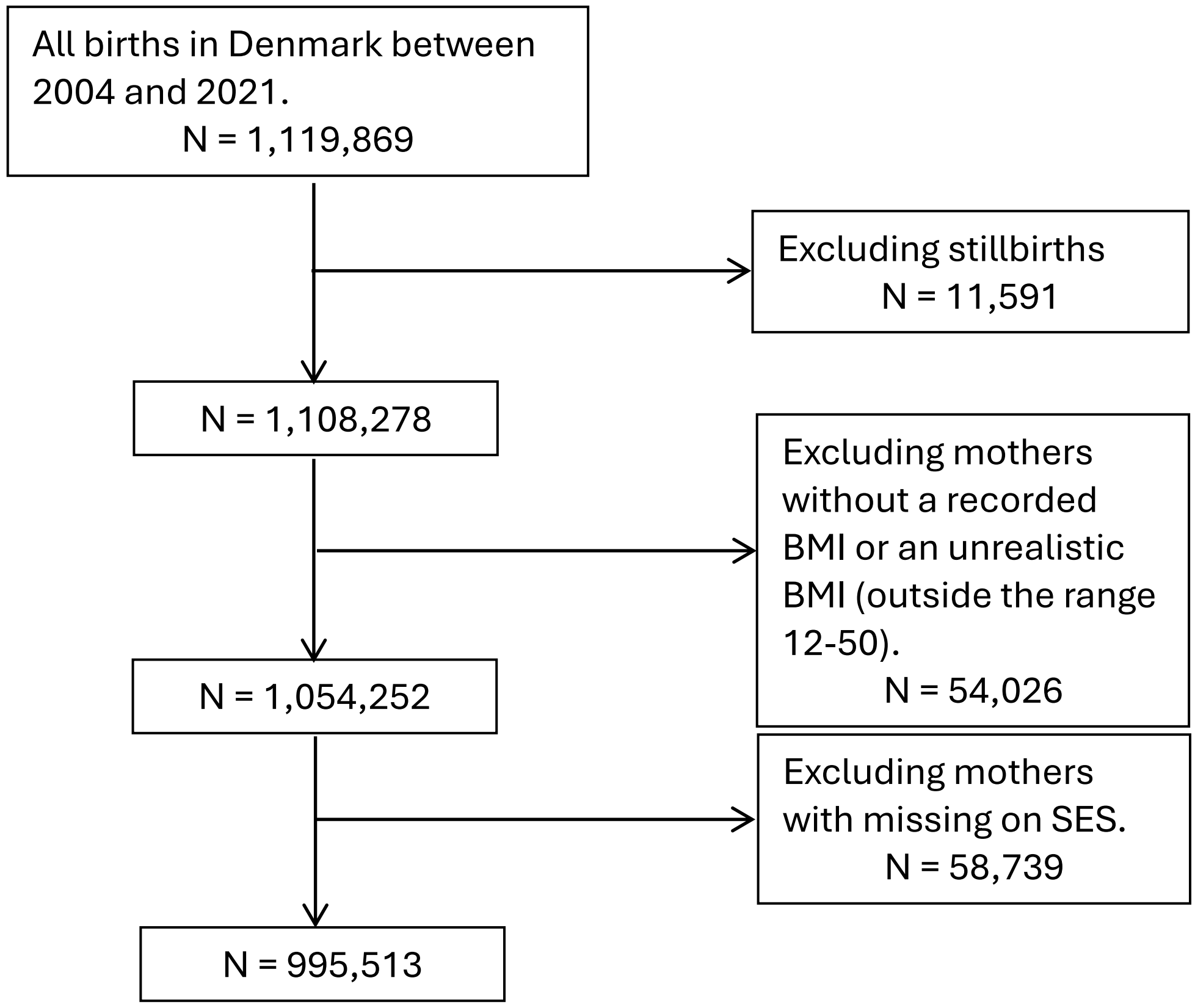

eTable 1. Odds ratios and 95% CIs for PPE restricted to primiparous women and stratified on psychiatric history.

	Psychiatric history		
	**None**	**Past**	**Recent**
**SES index**			
High SES	1.00 (ref)	1.00 (ref)	1.00 (ref)
Medium-high SES	**1.23 (1.15-1.31)**	**1.23 (1.13-1.35)**	**1.15 (1.09-1.21)**
Medium-low SES	**1.75 (1.64-1.88)**	**1.58 (1.44-1.74)**	**1.28 (1.21-1.35)**
Low SES	**1.86 (1.63-2.12)**	**1.74 (1.49-2.03)**	1.05 (0.96-1.15)
**BMI**			
Underweight	**1.36 (1.22-1.52)**	**1.25 (1.08-1.45)**	**0.87 (0.80-0.95)**
Normal weight	1.00 (ref)	1.00 (ref)	1.00 (ref)
Pre-obesity	1.06 (0.99-1.13)	0.99 (0.91-1.08)	**1.08 (1.03-1.13)**
Obesity class I	**1.16 (1.05-1.27)**	**1.14 (1.01-1.28)**	**1.12 (1.05-1.20)**
Obesity class II/III	**1.47 (1.31-1.65)**	**1.20 (1.03-1.40)**	**1.22 (1.13-1.33)**

eTable 2. Mediation analysis with SES as exposure, BMI as mediator, and PPE as outcome restricted to primiparous women. Stratified on psychiatric history

SES index	Direct effect	Indirect effect	Proportion mediated
**No psychiatric history**			
High → Medium-high	1.004 (1.002,1.005)	1.000 (1.000,1.000)	-0.55%
High → Medium-low	1.011 (1.010,1.013)	1.000 (1.000,1.000)	-0.04%
High → Low	1.013 (1.010,1.017)	1.000 (1.000,1.000)	1.79%
**Past psychiatric history**			
High → Medium-high	1.012 (1.006,1.019)	1.000 (1.000,1.000)	0.23%
High → Medium-low	1.030 (1.024,1.036)	1.000 (1.000,1.000)	0.50%
High → Low	1.038 (1.028,1.052)	1.000 (1.000,1.001)	1.15%
**Recent psychiatric history**			
High → Medium-high	1.030 (1.021,1.042)	1.001 (1.000,1.002)	2.96%
High → Medium-low	1.059 (1.046,1.073)	1.002 (1.001,1.002)	2.63%
High → Low	1.011 (0.993,1.030)	1.002 (1.001,1.003)	16.23%

eTabel 3. Proportion attributable risk percentage (PARP) for BMI and SES on PPE, restricted to primiparous women. Stratified on psychiatric history

	Psychiatric history		
	None	Past	Recent
BMI	5.2%	3.06%	3.45%
SES	20.1%	27.96%	11.93%

eTable 4. E-value for the indirect and direct effect

The lowest possible E-value is 1 (i.e. no unmeasured confounding is needed to explain away the observed association)

	Direct effect		Indirect effect	
**SES index**	**Estimate**	**CI**	**Estimate**	**CI**
**No psychiatric history**				
High → Medium-high	1.000	1.000	1.067	1.067
High → Medium-low	1.000	1.000	1.116	1.110
High → Low	1.033	1.000	1.116	1.098
**Past psychiatric history**				
High → Medium-high	1.000	1.000	1.143	1.122
High → Medium-low	1.033	1.000	1.218	1.202
High → Low	1.033	1.033	1.229	1.189
**Recent psychiatric history**				
High → Medium-high	1.033	1.033	1.198	1.148
High → Medium-low	1.047	1.047	1.272	1.233
High → Low	1.067	1.047	1.128	NaN
